# Survival Outcomes for Men over 80 Years Undergoing Transrectal Ultrasound-Guided Prostate Biopsy: A Prospective Analysis

**DOI:** 10.3390/cancers16233995

**Published:** 2024-11-28

**Authors:** Dareen Alghamdi, Neil Kernohan, Chunhui Li, Ghulam Nabi

**Affiliations:** 1 Division of Imaging Sciences and Technology, School of Medicine, Ninewells Hospital, University of Dundee, Dundee DD1 9SY, UK; 2 Radiology Department, College of Applied Medical Sciences, Imam Abdulrahman bin Faisal University, P.O. Box 1982, Dammam 31441, Saudi Arabia; 3 Department of Pathology, Ninewells Hospital, Dundee DD9 1SY, UK; neil.kernohan@nhs.scot; 4 School of Science and Engineering, University of Dundee, Dundee DD1 4HN, UK; 5 Division of Cancer Research, School of Medicine, Ninewells Hospital, University of Dundee, Dundee DD1 9SY, UK; g.nabi@dundee.ac.uk

**Keywords:** prostate cancer, PSA, transrectal ultrasound biopsy (TRUS), nomogram, prognosis

## Abstract

**Introduction:** Prostate cancer is the second most prevalent cancer among elderly males in Western countries. TRUS biopsy remains a standard diagnosing approach for prostate cancer but poses notable risks, particularly in older men, including complications such as sepsis, acute retention, and rectal bleeding, which can lead to substantial morbidity and mortality. This study aimed to evaluate cancer-specific survival outcomes in men aged over 80 years and whether there is any cancer-specific survival advantage for TRUS biopsy procedure. **Methods**: Between January 2005 and December 2015, we studied outcomes of 200 patients (median age, 82 years) with elevated prostate-specific antigen (PSA) levels (>4.0 ng/mL) and/or abnormal digital rectal examination (DRE) who underwent TRUS biopsy. Each participant was followed up until death using an electronic system and a unique identifier in a defined geographical area. Cancer-specific and overall survival analyses were carried out utilising SPSS, while R Project was employed to construct and evaluate two nomograms survival duration and predict the risk of death post-biopsy. All statistical tests were two-tailed, with significance set at *p* < 0.05. **Results**: Amongst the participants, only 24 patients were alive at the end of follow-up (median age, 91 years). The PSA levels ranged from 4.88 to 102.7 ng/mL. Log-rank and Breslow tests indicated that higher PSA levels, the development of metastases, and ISUP grade group 8–10 were associated with shorter survival times. Age, co-morbid conditions, and tumour type were incorporated into the nomogram due to their clinical significance. Patients aged <81 years had lower mortality risk, while those aged >88 years faced higher mortality risks. Complications from the biopsy increased mortality risks in both cancerous and benign cases, and metastasis significantly heightened the likelihood of death. However, co-morbid conditions did not influence survival probability. **Conclusions**: Our findings underscore that older age (specifically 80 years and above), high Gleason score, metastasis, and elevated PSA levels are predictive of poorer survival outcomes in elderly men following TRUS biopsy.

## Simple Summary

Prostate cancer is common among older men, and its diagnosis often involves a procedure called transrectal ultrasound (TRUS) biopsy, which helps detect cancer but also carries risks like infection and bleeding, particularly for elderly patients. This study evaluated the survival of men over 80 years of age to determine if undergoing a TRUS biopsy might improve their chances of surviving prostate cancer. By examining the records of 200 patients who had this biopsy, the study identified factors linked to poorer survival, including high levels of prostate-specific antigen, advanced cancer grade, and metastases. These findings can assist in a more accurate evaluation of the risks associated with TRUS biopsy in elderly patients and may guide future research toward developing safer or alternative strategies for diagnosing and managing prostate cancer in this population.

## 1. Introduction

Prostate cancer (PCa) is the most prevalent disease in elderly males and the second leading cancer in the Western countries [1]. The rate of prostate cancer has progressively increased during the previous ten years [1,2]. From 2000 and 2050, the number of men above the age of 65 is predicted to quadruple worldwide [2]. Prostate cancer incidence rates in males aged 65 and older have plateaued. Furthermore, it has been reported that the possibility of contracting prostate cancer rises from 0.005% in men below 39 years, 2.2% in men 40 to 59 years, and 13.7% in men 60 to 79 year. The recent lifetime probability of being diagnosed with prostate cancer is 16.7%, which is equivalent to 1 in every 6 men [3]. Histology studies suggest an even higher risk of prostate cancer; Carter and colleagues reported that 50% of males aged 70 to 80 years had histological evidence of prostate cancer [3,4]. 

Prostate-specific antigen (PSA) assay is the most effective screening test, which, when combined with digital rectal examination (DRE), significantly improves cancer detection rates [5]. The moderate incidence increases seen over the last decade are probably due to the increase in PSA testing among men under the age of 65 [3]. However, the PSA test has substantial limitations and potentially hazardous effects that outweigh the possible benefits of screening. According to Lin et al. [6], PSA false positives can cause psychological trauma in some men for up to a year after the test. Djulbegovic et al. [7] conducted a meta-analysis that included four additional trials, two of which were large, and discovered that PSA screening had no benefit in lowering prostate cancer mortality in the general population. Thus, although annual screening appears to reduce prostate cancer fatalities slightly, there is no substantial reduction in all-cause mortality in males under the age of 75 who have no risk factors for cancer or cardiovascular disease. Evidence also suggests that the DRE may not be associated with reducing death; however, it may lead to an increased number of false positives. This leads to unnecessarily invasive diagnostic tests, which can cause and contribute to pain, erectile dysfunction, urinary incontinence, and overdiagnosis and overtreatment for prostate cancer [8,9,10,11]. 

The most practised technique for detecting prostate cancer is a transrectal ultrasound-guided biopsy [12]. Recently, it has been reported that MRI can be used as a reliable diagnostic tool instead of performing prostate biopsy to detect prostate cancer, especially in patients who have undergone a previous negative biopsy, showing a comparable detection rate of clinically significant PCa [13,14]. Therefore, MRI may be used as an alternative as it is also considered a valuable tool in reducing over-diagnosis and overtreatment, especially in the context of active surveillance [15]. Additionally, MRI can guide targeted biopsies, improving the likelihood of detecting significant cancer and reducing sampling errors [16]. Furthermore, MRI guide targeted biopsies can help avoid the risks associated with systematic biopsies, such as bleeding and infection [15], while providing detailed imaging that aids in accurate staging and treatment planning. According to Carroll et al. [17], MRI has been proven to be low-risk for patients, making it a safer alternative in many cases. However, there are disadvantages to using MRI, including its high cost and the potential for patient discomfort or claustrophobia during the procedure [18]. 

Overall, TRUS biopsy is considered a generally well-tolerated process. It has been reported that more than 80% of men who received TRUS biopsy agreed to repeat the procedure under local anaesthesia if necessary [19]. However, TRUS biopsy is associated with risks such as discomfort, acute urine retention, haematuria, haematospermia, rectal bleeding, erectile dysfunction, and infection, as well as infectious complications [20]. According to decision-analytic models, for an older man or one with major co-morbid diseases, a competing hazard is considerably more likely to cause morbidity or mortality than prostate cancer [21], implying that more elderly men die with slowly advancing prostate cancer rather than from prostate cancer itself. Side effects from treatment are also more prevalent in older males [22]. These factors make them a more challenging population for prostate cancer screening and treatment. A study by Iwamoto et al. (2021) developed a screening strategy for significant prostate cancer in men over 75. The study concluded that increasing the PSA threshold to 12 ng/mL reduces unnecessary biopsies while accurately diagnosing clinically significant PCa cases. This study supports avoiding invasive procedures in men over 75 due to higher complication risks and limited survival benefits [23]. Despite the risks associated with bleeding and infection, TRUS biopsy remains widely utilised due to its accessibility and real-time imaging capabilities. Its role in detecting prostate cancer in elderly patients, particularly those aged 80 and above, is critical for evaluating cancer-specific survival outcomes. 

There is currently no age limit for offering patients a TRUS biopsy when they appear with a high PSA [24]. The recent European guidelines advocate active treatment for patients with a life expectancy of 10–15 years after diagnosis. These figures remain contentious, emphasising the need for an age cutoff as well as additional examinations of concomitant co-morbidities [24]. As previously mentioned, TRUS-guided biopsy leads to several complications, so patients should be advised of the risks before having one performed. This is particularly crucial for older patients aged above 70 years old, as complications such as sepsis, acute retention, and rectal bleeding can lead to considerable morbidity and mortality [20]. Studying prostate cancer and transrectal ultrasound (TRUS) biopsy in men aged over 80 is essential for several reasons. For instance, elderly men face substantial risks of prostate cancer, with elevated incidence and mortality rates within this demographic [25]. Elderly men undergo physiological alterations and acquire more co-morbid conditions, both of which can influence the decisions made about treatment and the subsequent outcomes for prostate cancer patients [26]. Moreover, making decisions about treatment for prostate cancer in older men is intricate, involving a delicate balance between the possible advantages of aggressive treatments and the risks of excessive treatment and associated health complications [27]. Therefore, investigating the effectiveness, safety, and impact on quality of life of TRUS biopsy approaches is vital for well-informed decision-making processes. To our knowledge, there is a lack of evidence as to whether or not TRUS biopsies in men more than 80 years of age improve cancer-specific survival. Men in this age group may be at a greater likelihood of being harmed by an invasive test rather than benefiting from this. As a consequence, the aim of our study was to evaluate cancer-specific survival outcomes in men aged over 80 years and whether there is any cancer-specific survival advantage for the TRUS biopsy procedure.

## 2. Materials and Methods

### 2.1. Study Approval and Ethics

This prospective cohort study was approved by NHS Tayside Ethics (Ref: IGTCAL11168) in June 2023, with prior institutional approval reference 70249. 

### 2.2. Study Population

The current prospective study was conducted between 1 January 2005 and 31 December 2015, aiming to evaluate cancer-specific survival outcomes in men aged 80 years and over, and whether there is any survival advantage for TRUS biopsy use.

Most patients suffered from a lower urinary tract and had non-specific symptoms as there was no prostate cancer screening programme implementation in this cohort. The lack of a universal screening programme may result in prostate cancer being diagnosed at a later stage, which could influence both survival outcomes and treatment decisions in elderly men.

The inclusion criteria were as follows:

❖ Men aged 80 years and older;
❖ Patients with elevated PSA levels (>4.0 ng/mL) or abnormal DRE, and men who underwent TRUS biopsy.

The exclusion criteria were as follows:

❖ Patients with an incomplete biopsy procedure, and PSA levels (<4.0 ng/mL);
❖ Patients were also excluded if there was insufficient data, such as incomplete biopsy results or clinical diagnosis of prostate cancer.

**Figure 1:**
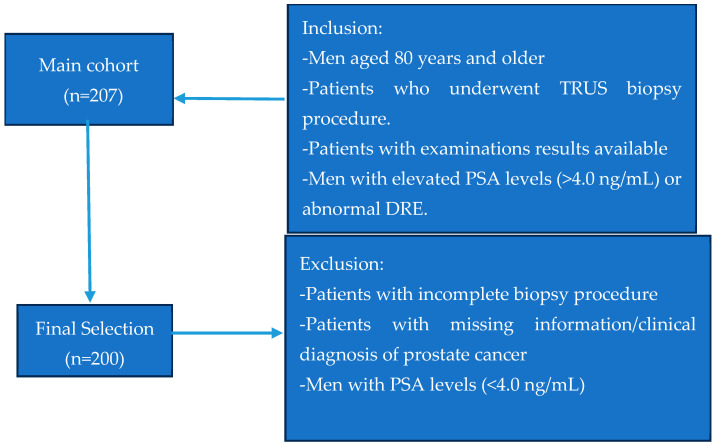
Flow diagram of patient selection.

A total of 200 consecutive men aged 80 years and over were included during the study period. They were scheduled for a prostate biopsy procedure. The selection process is illustrated in Figure 1. 

The primary outcome of the study was the cancer-specific survival rate of men aged 80 years and over who underwent transrectal-guided biopsy for suspected prostate cancer. 

#### Data Collection and Follow-Up

Data were collected, and each patient was followed-up with by using the data linkage methodology as described by our group previously. The diagnosis in all the cases was confirmed by a pathologist at Ninewells Hospital, NHS. During the follow-up period, patient outcomes were carefully tracked, with causes of death being meticulously documented through a thorough review of medical records or death certificates, providing a comprehensive and precise understanding of patient survival and mortality. All patients underwent 12-core TRUS-guided biopsies to ensure comprehensive sampling. Biopsy cores were obtained from the lateral, medial, and apical regions of the prostate under transrectal ultrasound guidance, following standard protocols for adequate tissue representation. The biopsy procedure was performed by experienced urologists, ensuring consistency in sample collection across all patients. Each biopsy core was immediately preserved for histopathological analysis.

This rigorous data acquisition process, combined with the deterministic data linkage using the unique Community Health Index (CHI) numbers, allowed for a robust and comprehensive analysis of patient outcomes thereby ensuring the integrity and completeness of the study. The use of CHI numbers enabled precise matching of patient data across various healthcare databases, facilitating the accurate tracking of survival outcomes, medical interventions, and mortality events throughout the follow-up period. This approach not only enhanced the reliability of the findings but also allowed for the inclusion of all relevant clinical data, minimising the risk of data omission or bias.

### 2.3. Data Analysis

Data were computed and analysed using the Statistical Package for the Social Sciences (SPSS, Version 28). Descriptive statistics were used to summarise the patients’ information. The statistical analysis focused on overall cancer-specific mortality, using methods such as the independent sample t-test and log-rank test to compare survival outcomes between two groups of patients diagnosed with cancer and those with benign tumours. Additionally, the Kaplan–Meier method, life table, and Cox regression calculations were applied to assess survival probabilities. 

Statistical code from the R Project (Version 4.0.3) was used to construct and assess two nomograms’ performance. The first nomogram aimed to quantify the risk of death, while a second dynamic nomogram was used to predict survival duration following the biopsy procedure.

The dynamic nomogram allowed for the determination of the parameters that influenced the accuracy of the model prediction. These parameters included complications, post-biopsy complications, patient age at the time of biopsy, the presence of metastasis, and co-morbid conditions. All statistical tests were two-tailed, with a statistically significant value defined as a *p* value of less than 0.05.

Subgroup analysis was conducted by dividing the patients into three groups based on survival probability to explore potential differences in predictive performance across different levels of risk. This approach allowed for the assessment of whether the nomogram performs similarly or differently in predicting survival outcomes for patients with varying levels of predicted risk.

Internal validation was performed using a hold-out approach, where 30% of the data was reserved as a validation set. On this validation set, receiver operating characteristic (ROC) curve analysis was conducted to evaluate the discriminatory ability of the nomogram. Additionally, the area under the receiver operating characteristic curve (AUC) was calculated for each subgroup to assess the stability of the predictive performance and whether the model’s accuracy varies under different conditions or patient characteristics. This internal validation procedure allowed us to evaluate the nomogram’s performance on unseen data from the same dataset used for nomogram development. 

## 3. Results

### 3.1. Patients’ Clinical Characteristics

The study included a total of 200 patients who met the inclusion criteria. Demographics and clinical characteristics of the cohort are presented in Table 1. The median age of the patients was 80 years, with 174 (87%) diagnosed with prostate cancer. At the time of data analyses, 24 patients with cancer were alive and 150 were dead. The median age for patients with cancer who were alive was 91 (IQR, 82–97 years), and the median age for patients with cancer who were dead was 88 (IQR, 81–99 years). 

Only 26 (13%) of the patients had a benign diagnosis. Ten patients with a benign tumour were alive and sixteen had died during follow-up. The median age for patients with benign tumours who were alive was 92 (IQR, 88–95 years old), while the median age for patients with benign tumours who were dead was 92 (IQR, 83–97 years old). 

**Figure A1:**
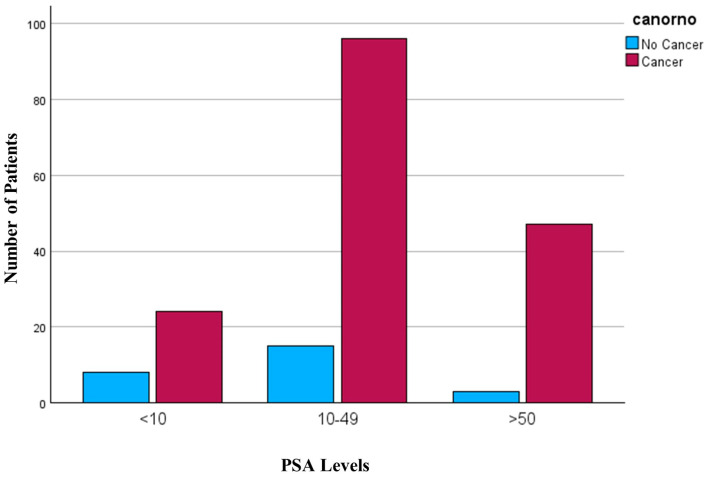
PSA levels for patients with and without cancer.

**Figure A2:**
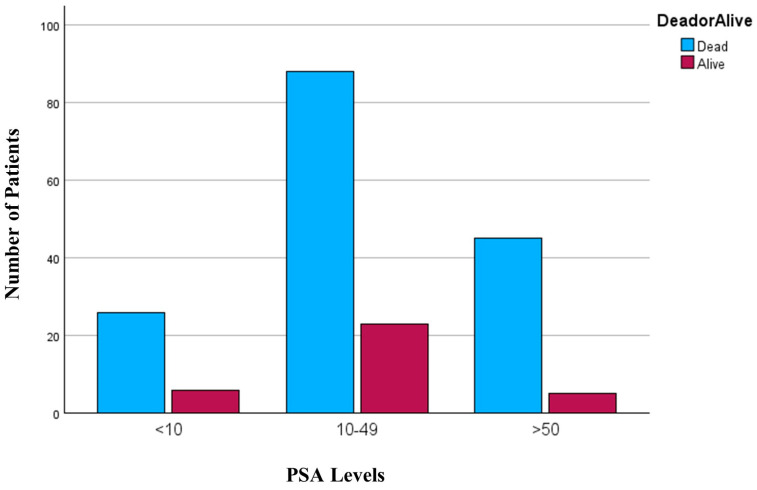
PSA levels for dead and alive patients.

The PSA levels ranged from 4.88 to 102.7 ng/mL, categorised as <10, 10–49, and >50. The total PSA level for all patients with cancer who were alive was 28.01 ± 23.05. Detailed PSA results are summarised in Figure A1 and Figure A2.

In addition, amongst the patients with benign tumours who were alive, 3 patients had PSA <10 and 7 patients had PSA > 50 (6.7 ± 3.54, 14 ± 2.18), respectively. The total PSA level for all patients with benign tumours who were alive was 11.81 ± 4.29. Moreover, amongst the patients with benign tumours who were dead, 5 patients had PSA <10, 9 patients had PSA 10–49, and only 2 patients had PSA >50 (4.88 ± 3.35, 22.41 ± 12.3, 102.7 ± 54.16), respectively. The total PSA level for all patients with benign tumours who were dead was 26.97 ± 34.92.

The ISUP grade groups were classified into three categories: 6, 7 and 8–10. Among all the cancer patients in the study, 18 had an ISUP grade group 6, 61 had an ISUP grade group 7, and 95 had an ISUP grade group 8–10.

The presence of metastasis was only reported amongst dead patients with cancer. A total of 12 patients with cancer who were dead from the total of 150 patients had metastases. Furthermore, a total of 12 live cancer patients had co-morbid conditions, whilst 12 had no co-morbid conditions. Regarding the dead cancer patients, 61 patients had co-morbid conditions and 89 had no co-morbid conditions. Four patients who were alive with benign tumours had a co-morbid condition and six had no co-morbid conditions. In addition, 6 patients who had benign tumours but were dead had co-morbid conditions and 11 did not have any co-morbid conditions. Biopsy complications were reported in 14 patients diagnosed with cancer and in 5 patients with benign diagnosis.

### 3.2. Survival Analysis

**Figure 2:**
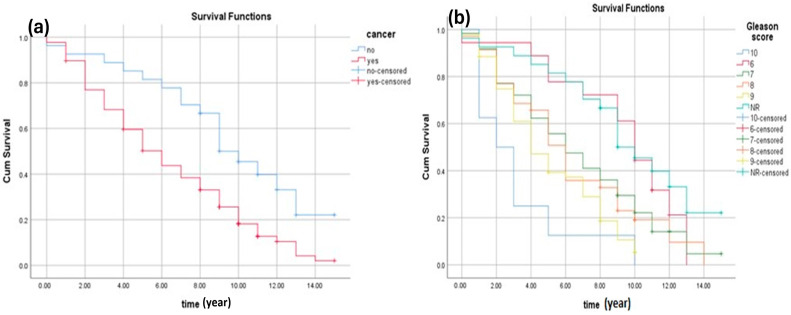
(**a**) The Kaplan–Meier survival estimates of prostate cancer. The estimated survival of benign patients was (8.5 ± 0.7, 95%CI 7.6–10.1), while that of cancer patients was (5.9 ± 0.3, 95% CI 5.4–6.5). (**b**) The survival rate of patients with cancer according to ISUP grade groups. The estimated survival of patients with GS 6 was (8.9 ± 0.8, 95% CI 7.3–10.5), for patients with GS 7 was (6.4 ± 0.5, 95% CI 5.5–7.4), and for patients with GS 8–10 was (4.6 ± 0.7, 95% CI 3.2–5.97).

For each group of interest, survival curves plots are an important aspect of survival analyses. The log-rank test represents the comparison of two groups (Table 2). The Kaplan–Meier test was used to estimate the probability of survival at a specified point in time. In the present study, there are two groups (patients with cancer and patients with benign tumours) that were compared to obtain the survival time (Figure 2a). The results in Figure 2a show that as expected the overall survival rate of patients with cancer (the red line) was significantly lower than patients without cancer (the blue line). This clearly shows that patients with no cancer had better survival.

Prostate cancer can be given an ISUP grade between 6 and 10. In practice, the lowest ISUP grade group is 6, which is a low-grade cancer. An ISUP grade group of 7 is considered intermediate-grade cancer, and an ISUP grade group of 8, 9, or 10 is high-grade cancer. Low-grade cancer grows more slowly and is less likely to spread than high-grade cancer. The result presented in Figure 2b suggests that patients with a low ISUP grade have a better chance of survival. The results in Figure 2b show that patients with 10, 9, and 8 ISUP grade (high-grade cancer) form the lowest survival rates. However, patients with 6 and 7 ISUP grade form the highest survival rates (lower-grade cancer). A very high ISUP grade group is regarded.

**Figure 3:**
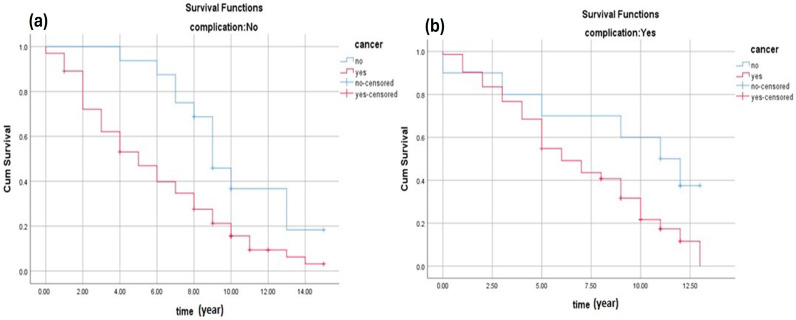
(**a**) The survival rate of patients without complications. The estimated survival of patients without complications and with benign tumours was (8.9 ± 0.6, 95% CI 7.6–10.1), while for patients with cancer, it was (5.5 ± 0.4, 95% CI 4.8–6.2). (**b**) The survival rate of patients with complications. The estimated survival of patients without cancer who experienced complications was (8.9 ± 1.4, 95% CI 6.0–11.6), while for cancer patients with complications, it was (6.5 ± 0.4, 95% CI 5.7–7.3).

Figure 3a shows the survival rate of patients who did not develop complications after the biopsy. The results clearly shows that patients without cancer have a better chance of survival. Figure 3b shows the survival rate of patients who developed complications after the biopsy. The results clearly show that patients without cancer have a better chance of survival although they had complications from the biopsy. 

**Figure 4:**
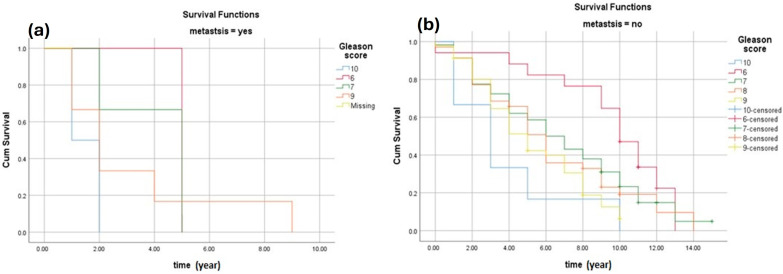
(**a**) The survival rate of patients with metastasis according to the ISUP grade groups. The estimated survival of patients with metastasis with a GS of 6 was (5 ± 0.0, 95% CI 5.0–5.0), for patients with a GS of 7 was (4.0 ± 1.0, 95% CI 2.0–5.96), and for patients with a GS of 8–10 was (2.4 ± 0.8, 95% CI 0.6–4.1). (**b**) The survival rate of patients with no metastasis according to the ISUP grade groups. The estimated survival of patients without metastasis with a GS of 6 was (9.1 ± 0.8, 95% CI 7.5–10.7), for patients with a GS of 7 was (6.6 ± 0.5, 95% CI 5.6–7.6), and for patients with a GS of 8–10 was (4.8 ± 0.8, 95% CI 3.3–6.4).

Figure 4a shows the survival rate of patients with metastasis according to their ISUP grade groups. The results show that patients with an ISUP grade group of 8, 9, and 10 had a lower chance of survival. As a result, it suggests that patients with a low ISUP grade have a better chance of survival even if they had metastasis. The results indicate that patients with 10, 9, and 8 ISUP grade group (high-grade cancer) form the lowest survival rates. However, patients with 6 and 7 ISUP grade groups form the highest survival rates (lower-grade cancer). Figure 4b shows the survival rate of patients with no metastasis according to their ISUP grade. The results clearly suggest that patients with a low ISUP grade have a better chance of survival. The results indicate that patients with 10, 9, and 8 ISUP grade group (high-grade cancer) form the lowest survival rates. However, patients with 6 and 7 ISUP grade groups form the highest survival rates (lower-grade cancer).

**Figure 5:**
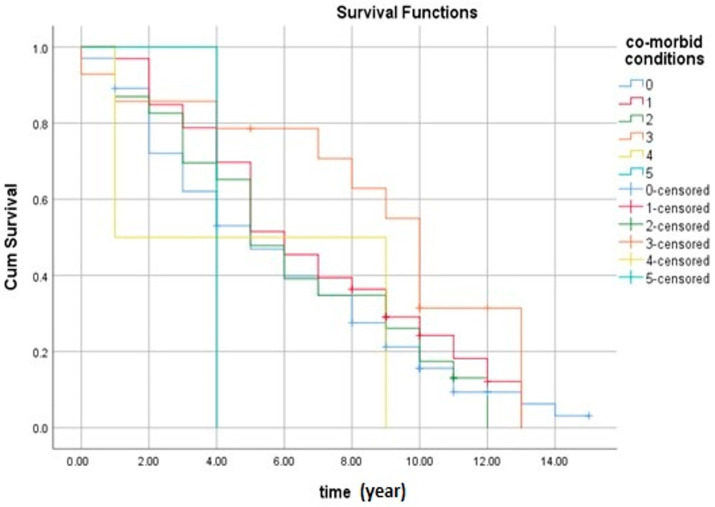
The survival rate of cancer patients according to the number of co-morbid conditions. The estimated survival rate of cancer patients according to the number of co-morbid conditions was (6.3 ± 0.2, 95% CI 5.8–6.8). Note: 0 = no conditions, 1 = 1 condition, 2 = 2 conditions, 3 = 3 conditions, 4 = 4 conditions, and 5 = 5 conditions.

Figure 5 shows the survival rate of patients with cancer according to number of co-morbid conditions they have. The results indicate that the number of co-morbid conditions was not associated with survival rate among cancer patients.

To examine the differences in the survival curves, the log-rank test was performed. The results presented in Table 2 show that in patients who lived <4 years, 4–8 years, and >8 years the survival rate was significantly lower in patients with benign tumours (*p* = <0.05). Ultimately, the survival rate was higher in patients without cancer. The results clearly show that patients with higher PSA, present metastasis, and an ISUP grade group 8–10 had lower survival time. However, the results also show that cancer was not the main cause of death in 62 out of the 150 patients who were diagnosed with cancer. Patients who did not die from cancer died from other conditions. Other patients died for unknown reasons. The causes of death were collected during patient follow-up through medical record reviews or death certificates.

### 3.3. Nomogram

**Figure 6:**
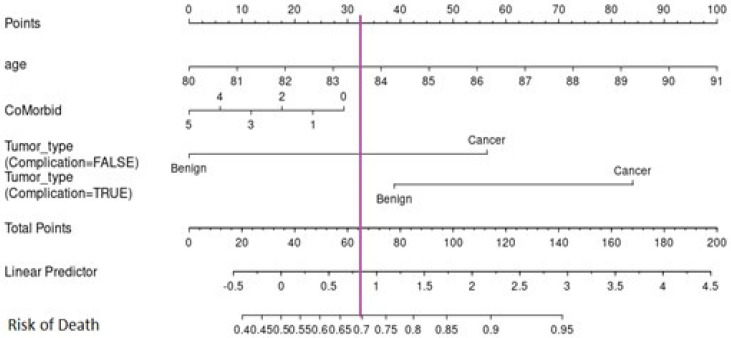
The nomogram for predicting the risk of death for patients with cancer at the age of biopsy.

**Figure A3:**
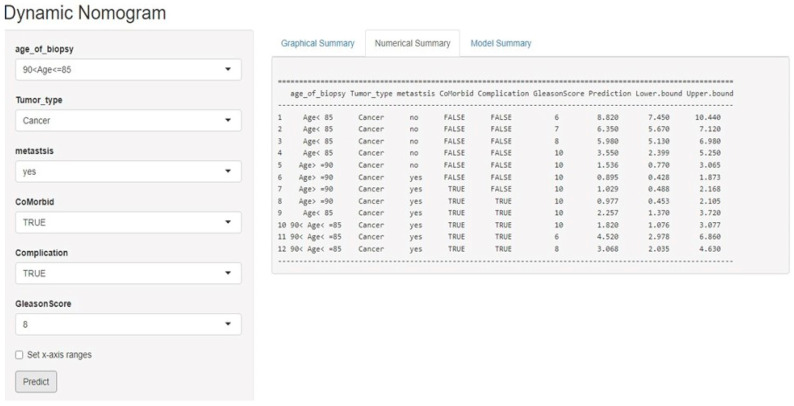
Predicting the survival probability for patients undergoing TRUS biopsy-diagnosed PCa.

To predict the risk of death for the cohort at the age of biopsy, a nomogram was constructed (Figure 6). The age, co-morbid conditions, and tumour grade groups were included in the nomogram as they were classified as clinically important. The risk of death was lower than 40% for those below 20 points and higher than 95% for those with over 140 points. The risk of death was lower amongst those aged below 81 years and higher amongst those aged above 88 years. The results also show that complications from the biopsy increased the risk of death for patients with cancer and benign tumours. Thus, the results clearly show that patients who suffered from complications after the biopsy had a lower survival rate even if they did not have cancer. The nomogram for this study can be accessed online to assist researchers and physicians via the following link: https://7qdqln-dareen-alghamdi.shinyapps.io/dynamic/ (for additional details, refer to Figure A3). The predicted survival probability over time could be easily calculated by inputting clinical features and reading the webserver’s output figures and tables. 

The results presented in Figure 6 shows that in patients who lived less than 4 years, the survival probability was lower than 0.2 for those below 100 points and 0.95 for those higher than 270 points. In addition, in patients who lived 4 to 8 years, the survival probability was lower than 0.05 for those below 100 points and 0.8 for those higher than 260 points. Regarding patients who lived more than 8 years, the survival probability was lower than 0.05 for those below 120 points and 0.7 for those higher than 270 points. The results indicate that the probability of death increased with age.

**Figure 7:**
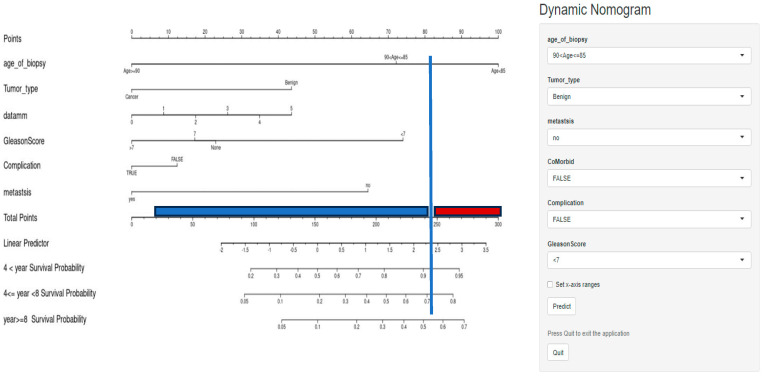
Dynamic nomogram for predicting survival rate. Risk classification: blue line = low-risk, red line = high-risk.

As expected, the probability of death was lower among patients with benign tumours and higher among cancer patients. The results also show that the survival rate is lower among patients who developed complications from the biopsy. The results also show that metastases increased the probability of death. However, survival probability was not affected by co-morbid conditions. As presented in Figure 7, patients were classified into low-risk (score ≤ 240) or high-risk (score > 240).

**Figure 8:**
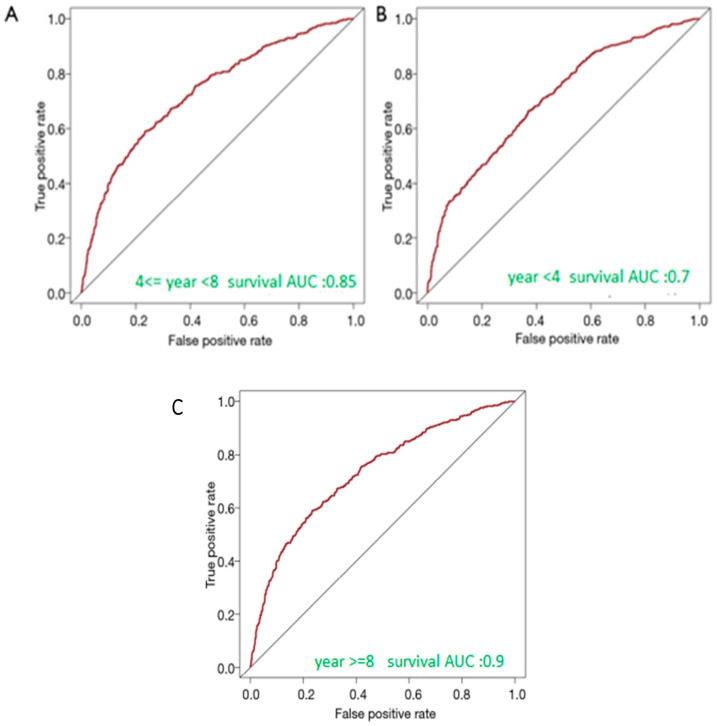
The ROC curves of the nomogram predicting the risk of death in cancer patients. (**A**) Predicting: year < 4. (**B**) Predicting: 4 ≤ year < 8. (**C**) Predicting: year ≥ 8. AUC refers to the area under the receiver operating characteristic curve.

The survival AUC of the prediction model presented in Figure 8 was 0.85, 0.70, and 0.9 in patients who lived <4 years, 4–8 years, and >8 years, respectively.

## 4. Discussion

### 4.1. Key Findings of Study

The study specifically assessed the survival benefits of performing a transrectal prostate biopsy in men aged 80 years and older. The findings demonstrated a high prevalence of prostate cancer (87%; 174/200), which is not unexpected. However, a significant proportion of men died from unrelated causes during the follow-up. The study analysed various factors influencing survival outcomes in this demographic, including the International Society of Urological Pathology (ISUP) grade, co-morbid conditions, and age. 

The inclusion criteria of our study were men aged 80 years and older and men with elevated prostate-specific antigen (PSA) levels (>4.0 ng/mL). The PSA levels of all included participants ranged between 4.88 and 102.7. Nevertheless, as mentioned earlier, PSA has substantial limitations and potentially hazardous effects that outweigh the possible benefits of screening. For instance, the high rate of false positives limits the utility of PSA screening [6], which may assist just a limited proportion of men and can vary with age and ethnicity, leading to challenges in setting universal reference ranges [28]. Thus, it has not benefitted in lowering prostate cancer mortality in the general population [7].

Transrectal ultrasound-guided (TRUS) needle biopsy remains the preferred diagnostic method for prostate cancer due to its safe and routine performance in an out-patient environment, typically in the office. However, TRUS biopsy does not come without complications. According to the study’s result, biopsy complications were reported in only 1 live patient with cancer, 13 deceased patients with cancer, 1 live patient with a benign tumour, and 4 deceased patients with benign tumours. Notably, complications from the biopsy increased the risk of death for patients with cancer and benign tumours. The results clearly indicate that survival rate is lower among patients who developed complications from the biopsy. 

The study findings demonstrate that the prediction model’s performance, as indicated by the survival AUC, varies across different survival timeframes for men over 80 years undergoing transrectal-guided biopsy. The model shows high discriminatory power for patients who lived less than 4 years (AUC = 0.85) and more than 8 years (AUC = 0.90), indicating its reliability in predicting survival outcomes at these extremes. However, it has moderate accuracy for the 4–8-year survival group (AUC = 0.70). Overall, the high AUC values highlight the model’s strong predictive capabilities, offering valuable prognostic insights that could enhance clinical decision-making and patient management strategies.

The study also revealed that the overall survival rates were significantly lower in patients with cancer compared to those with benign tumours. Patients with cancer were censored in the analysis, reflecting poorer survival outcomes compared to those without cancer. Thus, the probability of death was lower among patients with benign tumours and higher among patients with cancer. Specifically, survival rates across all survival timeframes (<4 years, 4–8 years, and >8 years) were significantly higher in patients with benign tumours (*p* < 0.05). Metastasis was reported only in deceased cancer patients; 12 of 150 deceased cancer patients had metastases, which, as anticipated, increased the probability of death. 

Age is a major risk factor for cancer. The risk of death was lower amongst those aged below 80–81 years and higher amongst those aged 88 years or older. The main cause of death in our study was not necessarily prostate cancer, and complications from other diseases are a major concern during prostate cancer treatment. Patients with co-morbidities typically undergo non-aggressive treatment [29,30,31]. However, the number of co-morbid conditions was not associated with survival rate among cancer patients. Thus, survival probability was not affected by co-morbid conditions. Men without any accompanying health conditions face similar risks of mortality from prostate cancer, compared to men with two or more co-morbidities, who are significantly more likely to die from other medical conditions other than prostate cancer [21].

The Gleason score (GS) was correlated with a higher probability of death amongst prostate cancer patients. The GS in this study was classified into three groups based on the International Society of Urological Pathology (ISUP) as follows: 6, 7, and 8–10. Amongst the cancer patients who were alive, 4 patients had an ISUP grade of 6, 8 patients had an ISUP grade of 7, and 12 patients had an ISUP grade group of 8–10. Moreover, amongst the cancer patients who were dead, 14 patients had an ISUP grade of 6, 53 patients had an ISUP grade of 7, and 83 patients had an ISUP grade of 8–10. These findings reaffirm that patients with a low Gleason score have a better chance of survival compared to patients with 8, 9, and 10 ISUP grades (high-grade cancer) form the lowest survival rates, which is indicative of aggressive cancer. Conservative treatment for aged individuals yielded acceptable results with a Gleason score of 6–7, whereas patients with a score of 8–10 had a poor prognosis [32].

### 4.2. Comparison of Findings to the Reported Literature

Prostate cancer cases have risen globally over the last 30 years, with a dramatic increase observed in the United Kingdom [33]. The frequency of prostate cancer in the UK was 32 per 100,000 in 1975; it rose to 95 per 100,000 in 2005 [34]. The incidence rate is predicted to increase by 15% between 2023 and 2025 and 2038 and 2040, with an estimated 85,100 new cases in the future [35]. It has been reported that 60% of cases are diagnosed in men above 70 years of age, with the majority falling between the ages of 70 and 79 years [34]. However, prostate cancer-specific survival rates have improved to over 20 years, primarily due to the discovery of a higher proportion of latent, early-stage, slow-growing cancers [34]. In England, the survival rate of men diagnosed with prostate cancer was 10 or more years in 2013–2017 [35]. The increase in survival rate is primarily because of early treatment in younger patients, who usually have a longer life expectancy and a general increase in longevity [36]. According to the Prostate, Lung, Colorectal, and Ovarian Cancer (PLCO) research and the European Randomised research of Screening for Prostate Cancer (ERSPC), prostate cancer screening studies show that there is an enhancement in disease-specific survival, yet still no substantial influence on overall survival rate [37,38]. Regarding clinical practice, the identification of prostate cancer is based on a combining of PSA, DRE, and TRUS biopsy [39]

The normal PSA level for the elderly was debated; the generally accepted limit of 4 ng/mL may not apply to this age group [8]. PSA levels of 20 ng/mL and even 30 ng/mL have been suggested as potentially more confirmative in the older population [34,40,41]. A PSA result of “10 ng/mL” has been demonstrated to be associated negatively with cancer in the elderly, prompting calls for a higher PSA cutoff limit [34,42]. 

A Brazilian study found that up to 75% of patients experienced haemorrhagic problems [43]. These included haematuria (56.3%), rectal bleeding (32.3%), and hematospermia (21.8%) [43]. Minor infectious issues such as urinary tract infections (UTIs) and prostatitis were reported in 14.4%, whereas serious complications such as sepsis, gross haematuria, and urine retention were reported in 2.9% [43]. Similar findings were reported in numerous additional trials, with haematuria, discomfort, rectal haemorrhage, and UTI being the most prevalent consequences [44,45]. Osama et al. [46] also reported that complications following the TRUS biopsy procedure may encompass various issues such as bleeding from the urethra, blood in semen (hematospermia), bleeding from the rectum, difficulty in emptying the bladder (urinary retention), urinary tract infections, infectious complications, challenges with erectile function, and pain. All these complications may increase the risk of death in elderly patients. Thus, prior to conducting a TRUS biopsy procedure, all patients should be adequately advised about the rare but substantial severe consequences, such as urosepsis and urine retention [44,45]. Although TRUS biopsy is not recommended in some guidelines due to risks such as bleeding, infection, and sepsis, it remains widely used for prostate cancer diagnosis. Recent studies suggest that MRI can serve as a reliable alternative to prostate biopsy for detecting prostate cancer, especially in cases with a previous negative biopsy, and offers similar detection rates for significant cancer [13,14]. Nonetheless, MRI is associated with a high cost and potential discomfort or claustrophobia [18]. 

The routine use of antibiotic prophylaxis has led to lower infection rates, although the practice varies between units from a single dosage to a three-day course [43,47,48,49]. In addition, clinical problems and hospitalizations following a TRUS prostate biopsy have risen over the previous decade, owing mostly to an increase in infection rates. Nam et al. [50] found that in a population-based analysis of 75,190 males who underwent transrectal prostate biopsy infections accounted for 71.6% of hospital admissions, followed by bleeding in 19.4% and urine retention in 9.0%. Loeb et al. [51] found fever in 4.2% of the 10,474 transrectal biopsies conducted in the European Randomized Study of Screening for Prostate Cancer (Rotterdam portion) and hospitalisation in 0.8% of the patients. Carignan et al. [52] found that in 5798 patients who underwent TRUS biopsy, there was an increased risk of infection, which increased from 0.52% in 2002–2009 to 2.15% in 2010–2011. Approximately 52% of the infections caused by pathogens (Escherichia coli in 75%) resistant to ciprofloxacin, particularly in men with diabetes, chronic obstructive pulmonary disease, and patients who were in hospital during the previous month. Moreover, Pinkhasov et al. [53] found that 2.5% of 1000 people who underwent transrectal prostate biopsy required hospitalisation due to urosepsis in 1.2%, urine retention in 0.8%, and gross haematuria in 0.4%. Loeb et al. [52] discovered in a random sample of Medicare participants (Surveillance, Epidemiology, and End Results) from 1991 to 2007 that prostate biopsy was correlated with a 2.65-fold increased risk of hospitalisation due to infections within 30 days compared to the control population.

Consistent with this, Klemann et al. [54], studying older men with median age 67 years, reported that men with benign initial biopsy results had a lower risk of prostate cancer-specific mortality. Their results also revealed that patients with benign first biopsy results were at an increased risk of dying from reasons other than prostate cancer. According to their study, only 287 of the 3056 men who received PSA screening and had a benign initial TRUS biopsy result were diagnosed with prostate cancer, where 7 died from the disease after 11 years of follow-up. After 14 years, patients with benign initial biopsies had projected progression-free survival and prostate cancer-specific survival rates of about 80% and 97%, respectively. 

According to the Japanese Government Lifetable [55], the average projected time of life remaining for males aged 80 is 8.8 years. Pneumonia is the leading cause of mortality in older men, followed by malignant neoplasms, heart disease, and brain vascular disease, and the reasons for death in the current patients were consistent with those observed in the Japanese male population. Patients who died of causes other than prostate cancer seemed to have a comparable life expectancy as the overall population of senior men. This is consistent with our findings, which revealed that co-morbidities do not affect the survival rate or increase the mortality risk of patients with prostate cancer.

According to Bernard et al. [56], men over 75 years have a shorter restricted mean survival time (RMST) for death from all causes, including prostate cancer. At 5 years, males aged ≥75 years died 4.8 months earlier than those aged ≤54 years, with prostate cancer being the leading cause approximately 7 months earlier. The relation between age at diagnosis with prostate cancer-specific mortality (PCSM) was independent of other factors, including the Gleason score. According to their results, men aged ≥75 years died of prostate cancer at a rate of 1.36 to 1.49 times higher than men aged ≤54 years [56]. In general, these results provide additional indications that men aged above 75 years with metastatic prostate cancer suffer from unfavourable complications, possibly as a result of the faster progress of resistant disease, frailty, or co-morbidities, as well as their reduced capability to access treatments such as chemotherapy [56]. Scosyrev et al. [57] reported that older males are prone to be diagnosed with a new metastatic illness and die from prostate cancer. This is consistent with registry data demonstrating that males aged ≥75 years have poorer survival rates and quicker time to castration resistance than younger men [58]. In addition, with the current trend toward decreased PSA screening and an increase in males presenting with metastatic disease, the rate of new metastatic prostate cancer in older men may rise further [59]. 

### 4.3. Limitations of the Study 

The current study is subject to several limitations. Firstly, the study’s dependence on a single institution and a specific region for data collection adds institutional bias, which may restrict the findings’ generalisability. Thus, the study findings cannot be generalised to all elderly men over 80 years old undergoing TRUS. However, external validation was not conducted for this study due to a lack of access to external data. Secondly, the small sample size may limit the study’s statistical power and capacity to make meaningful results. Multivariable Cox proportional hazards regression was not utilised in this study due to concerns about overfitting associated with the limited sample size. We recommend that future research address this limitation by employing this method or external validation techniques with larger datasets to enhance the understanding of survival outcomes. Thirdly, the study did not account for all potential confounding factors, such as socioeconomic status or access to healthcare services, which may influence survival outcomes in elderly men. Thus, suggesting that future studies could include these variables or use alternative analytical methods to minimise the impact of confounders shows a proactive approach towards improving the robustness of future research in this area. Fourthly, selection bias is also a limitation of this study, especially since the study sample does not adequately represent all elderly men’s TRUS. Lastly, relying primarily on TRUS as a diagnostic tool may result in overlooking other viable diagnostic modalities or missing nuanced data that could influence the study’s outcomes and conclusions. These limitations highlight the importance of cautious interpretation and identify topics for future research to minimise potential biases and enhance the precision of study findings. 

## 5. Conclusions

The overall survival rate of patients with cancer was significantly lower compared to those with benign conditions. Our results indicate that patients with a high Gleason score and elevated PSA levels had a lower chance of survival. In addition, patients with metastasis had a lower chance of survival. However, the survival probability was not affected by co-morbid conditions. We recommend further research over a longer follow-up period to assess long-term survival outcomes in men over 80 who undergo transrectal-guided biopsy. Such studies would provide a comprehensive understanding of the long-term impact of this intervention on survival. Moreover, we suggest expanding the study to include a comparative analysis of various biopsy techniques, such as transperineal biopsy, to identify the most effective and least invasive method for this age group.

## Figures and Tables

**Table 1:** Patients’ clinical characteristics (n = 200).

		Cancer or Benign			
		Cancer		Benign	
		Alive	Dead	Alive	Dead
Patients (n)		24	150	10	16
Age: Median age range YR ≥ 80		91 (97–82)	88 (99–81)	92 (95–88)	92 (97–83)
Age of patient at biopsy: Median age range YR ≥ 80		81 (88–80)	82 (91–80)	80 (84–80)	83 (89–80)
PSA Categories	PSA < 10	n = 3 (5.54 ± 3.61)	n = 20 (6.58 ± 1.62)	n = 3 (6.7 ± 3.54)	n = 5 (4.88 ± 3.35)
	PSA (10–49)	n = 16 (19.54 ± 8.72)	n = 81 (23.92 ± 10.8)	n = 7 (14 ± 2.18)	n = 9 (22.41 ± 12.3)
	PSA ≥ 50	n = 5 (68.60 ± 6.11)	n = 49 (126 ± 62.65)	−	n = 2 (102.7 ± 54.16)
	Total	28.01 ± 23.05	51.48 ± 59.66	11.81 ± 4.29	26.97 ± 34.92
ISUP grade groups (n)	NR	−	−	26	
	6	4	14	−	
	7	8	53		
	8–10	12	83		
Metastasis: Yes		−	12		
Co-morbid conditions	Yes	12	61	4	6
	No	12	89	6	11
Complication	Yes	1	13	1	4

**Table 2:** Log-rank (Mantel–Cox) and Breslow (generalised Wilcoxon) to show comparison tests for survival rates.

		Years of Survival from Date of Biopsy		
		<4 Years	4–8 Years	>−8 Years
**Benign**	Number of Deaths	2	5	9
	PSA	77.35 ± 90.01	15.62 ± 12.42	22.08 ± 21.31
**Cancer**	Number of Deaths	55	51	44
	PSA	76.54 ± 80.16	41.60 ± 37.72	31.97 ± 36.92
	Number of Deaths due to prostate cancer	31	20	11
	Metastasis: Yes	7	4	1
	ISUP grade groups (n):			
	6	1	4	9
	7	17	19	17
	8–10	37	28	10
***p*** **-value**		0.0	0.0	0.0

## Data Availability

The data presented in this study are available within this article.

## Abbreviations

The following abbreviations are used in this manuscript:

**PCa**	Prostate Cancer
**PSA**	Prostate-specific antigen
**TRUS**	Transrectal ultrasound
**DRE**	Digital Rectal Examination
**ISUP**	International Society of Urological Pathology
**ROC**	Receiver operating characteristic
**AUC**	Area under the curve
**CHI**	Community Health Index
**GS**	Gleason score
